# Effect of extremely low frequency magnetic fields on oxidative balance in rat brains subjected to an experimental model of chronic unpredictable mild stress

**DOI:** 10.1186/s12868-021-00656-x

**Published:** 2021-09-06

**Authors:** Leticia R. Quesnel-Galván, Patricia V. Torres-Durán, David Elías-Viñas, Leticia Verdugo-Díaz

**Affiliations:** 1 Laboratorio de Bioelectromagnetismo, Departamento de Fisiología, Facultad de Medicina, Universidad Nacional Autónoma de México, Circuito Escolar s/n, Cuidad Universitaria, C.P.04510 Mexico City, Mexico; 2 Departamento de Bioquímica, Facultad de Medicina, Universidad Nacional Autónoma de México, Circuito Escolar s/n, Cuidad Universitaria, C.P.04510 Mexico City, Mexico; 3 Departamento de Ingeniería Eléctrica, Sección de Bioelectrónica, CINVESTAV, IPN, C.P.07360 Mexico City, Mexico

**Keywords:** Antioxidant system, Magnetic fields, Mild stress, TBARs

## Abstract

**Background:**

There has been an increasing interest in researching on the effects of extremely low-frequency magnetic fields on living systems. The mechanism of action of extremely low-frequency magnetic fields on organisms has not been established. One of the hypotheses is related to induce changes in oxidative balance. In this study, we measured the effects of chronic unpredictable mild stress induced-oxidative balance of rat’s brain exposed to extremely low-frequency magnetic fields.

**Methods:**

A first experiment was conducted to find out if 14 days of chronic unpredictable mild stress caused oxidative unbalance in male Wistar rat’s brain. Catalase activity, reduced glutathione concentration, and lipoperoxidation were measured in cerebrum and cerebellum. In the second experiment, we investigate the effects of 7 days extremely low-frequency magnetic fields exposure on animals stressed and unstressed.

**Results:**

The main results obtained were a significant increase in the catalase activity and reduced glutathione concentration on the cerebrum of animals where the chronic unpredictable mild stress were suspended at day 14 and then exposed 7 days to extremely low-frequency magnetic fields. Interestingly, the same treatment decreases the lipoperoxidation in the cerebrum. The stressed animals that received concomitant extremely low frequency magnetic fields exposure showed an oxidative status like stressed animals by 21 days. Thus, no changes were observed on the chronic unpredictable mild stress induced-oxidative damage in the rat’s cerebrum by the extremely low-frequency magnetic field exposure together with chronic unpredictable mild stress.

**Conclusions:**

The extremely low-frequency electromagnetic field exposure can partially restore the cerebrum antioxidant system of previously stressed animals.

## Background

The universal distribution of electronic devices and their relationship with health care issues has raised increasing interest in investigating the effects of extremely low frequency magnetic fields (ELF-MF) on health. Recently, several epidemiological studies report a carcinogenic effect in residential and occupational exposition to ELF-MF [1, 2]. Therefore, a guideline for the prevention, diagnosis, and treatments against diseases caused by ELF-MF has been published [3]. In contrast, there are experimental and clinical studies that support the beneficial effects of ELF-MF exposure [3–6].

The mechanism of action of ELF-MF in biological systems has not been established. There are several hypotheses to explain it, which have been explored experimentally [7]. Various effects of ELF-MF have been observed, like inflammation, heat shock protein induction, DNA damage, changes in signal transduction involved in the genesis of cancer, and apoptosis induction [8]. There is one possible common cause that can elicit those effects: changes in oxidative balance [9]. Some investigations concerning this hypothesis have been carried out in vitro [10] and in vivo [11–13].

In rats, the effects induced by ELF-MF differ because there are variations in the intensity, frequency, and duration of the stimulus [12], and the age of the animals [14]. However, one variable that is important to consider in the effect of ELF-MF is the metabolic state of the animals, which has been poorly explored.

There is experimental evidence that different types of stress produce different effects on the oxidative balance system [15]. It is known that Chronic Unpredictable Mild Stress (CUMS) generates an oxidative unbalance [16]. Our laboratory has found that acute restriction stress generates an increase in lipoperoxidation and oxidative damage to the brain. ELF-MF exposure together with restriction stress increases both parameters [17]. The present study investigates the effects of CUMS induced-oxidative balance of rat’s brain exposed to ELF-MF. Our research explores the interaction between CUMS and ELF-MF exposure to assess if mild stress influences the response to ELF-MF.

## Materials and methods

All procedures were conducted in accordance with the official Mexican norms NOM-051-Z00-1995 and NOM-062-ZOO-1999. The experimental protocols were approved by institutional Local Committee of Research and Ethics of Faculty of Medicine and conducted under the authority of the Investigation Division, Faculty of Medicine, UNAM Project License (085/2016).

### Animals

A total of 60 male Wistar rats aged 8 weeks (180–200 g) from the animal house facility of the Faculty of Medicine, UNAM, Mexico was used. To avoid other stressors in addition to those induced by the CUMS model, the rats, being gregarious animals, were housed in transparent boxes (2 rats/cage) that allowed them to see their congeners. Furthermore, the rats were always handled by only two people during all the processes. To reduce animal suffering, hygiene, safety, and environmental control measures were kept. The animal center maintained constant humidity of 60%, the temperature of 22 °C, and air ventilation, with a 12 h light-dark cycle, and with *ad libitum* access to rodent chow and water. The number of animals for each group (n) was decided based on previous studies and thus avoided the excessive number of rats [17]. The rats were adapted to the laboratory conditions for 4 days. Exclusion criteria if the animals presented a sickness or an infection of any type.

### Experimental model chronic unpredictable mild stress (CUMS)

CUMS model consisted of applying a different kind of mild stressor each day for 21 days [18]. The stressors were: movement restriction for 2 h, 4 °C temperature exposure, the combination of both, forced swimming for 15 min, 45° cage tilting for 2 h, food deprivation for 24 h, water deprivation for 18 h, and isolation for 48 h. These were applied in a random form.

### ELF-MF exposure

ELF-MF was applied with a device previously used in our laboratory [17]. Whole body of animals was exposed, placed in the center of two Helmholtz coils (internal diameter of 30 cm and separated by 30 cm). Coils were connected in parallel to a 120 V adjustable transformer (Staco Energy Products). Sinusoidal magnetic (60 Hz) flux density was 2.4 mT, which was measured using a hand-held Gauss/Tesla Meter (Alpha-Lab). The magnetic field background level was 50 µT. The temperature between the coils was monitored using a Hygro-thermometer (Extech Instruments), which remained constant during the 2 h of exposure (12:00–14:00 h).

### Experiment I

An experiment was done to identify that the CUMS model was established before ELF-MF exposure, where the stress was applied for 14 days with its respective control. The CUMS was applied to 6 rats for 14 days to assess if they expressed oxidative stress (CUMS14) and the control group of 6 rats for 14 days (C14). Rats were euthanized at day 15 and cerebrum and cerebellum were analyzed for oxidative balance.

### Experiment II

The experiment was carried out for 21 days. Six groups of 8 animals each were formed (Table 1).

Group 1. Control group for the whole 21 days period (C).

Group 2. Another control group was formed to find out if magnetic fields affect the oxidative balance of unstressed animals. For the first 14 days, the animals were in a control condition, and for the seven last days, they received ELF-MF exposure (C + MF).

Group 3. Stressed group (CUMS) for the whole 21 days period to compare with the other CUMS groups and with the control group.

Group 4. To find out if ELF-MF exposure together with the stress affects the oxidative balance, a CUMS + MF group was formed. CUMS was applied for 21 days and during the third week the rats also received ELF-MF exposure (CUMS + MF).

Group 5. To find out if the interruption of CUMS alters the effect of ELF-MF exposure on oxidative balance, another group was formed. Rats only received for 14 days CUMS, and then ELF-MF exposure during the next 7 days (preCUMS + MF).

Group 6. Finally, a physiological control group was formed with animals that received CUMS for 14 days, and the next 7 days they remained without any treatment (i.e., the rat cages were placed in between the coils, but the apparatus was shut off, preCUMS + Sham).

Rats were euthanized at day 22 and cerebrum and cerebellum were analyzed for oxidative balance.

**Table 1:** Groups of experiment II

Group	Banner (n = 8)	Treatment
1	C	Control condition for 21 days
2	C + MF	Control condition for 14 days and then for 7 days ELF-MF exposure
3	CUMS	Chronic Unpredictable Mild Stress for 21 days
4	CUMF + MF	CUMS for 21 days and the last 7 days they also received ELF-MF exposure
5	preCUMS + MF	CUMS for 14 days and then for 7 days ELF-MF exposure
6	preCUMS + Sham	CUMS for 14 days and the next 7 days without any treatment

### Body weight control

The animals were weighed once a week to register the percentage of gain at the end of the experiment.

### Plasma corticosterone levels

Plasma corticosterone concentration was quantified using an ELISA kit (Enzo Life Sciences), and a microplate reader Stat Fax 3200 (Awareness Technology Inc.) according to the manufacturer ´s instructions.

### Euthanasia and tissue preparation

The average final weight was 300 g per rat. Before decapitation, the animals were anesthetized with an intraperitoneal injection of sodium pentobarbital (200 mg/kg) [19]. The heads dissected, brains were removed and immediately separated into the cerebellum and cerebrum. Each area was stored and kept frozen at − 70 °C until the moment of their analyses.

### Oxidative stress measurements

One investigator was aware of the stress treatments, but in the moment of the data analysis a second investigator did not know to what group the samples belonged. If a negative absorbance was recorded during the determination of enzyme activity, it was removed. But then we used an extra sample.

All chemicals were of analytical grade. Hydrogen peroxide (H_2_O_2_), GSH, 5,5ʹ-dithiobis-(2-nitrobenzoic) acid (DTNB), and Na_2_HPO_4_ phosphate buffer (pH 7.4), trichloroacetic acid (TCA), tetraethoxypropane (TEP) were purchased from Sigma Aldrich, Saint Louis, Missouri, USA.

### Catalase enzyme activity

The determination of catalase (CAT) activity was based on the decrease in the absorbance of hydrogen peroxide as the reaction substrate. CAT activity in the supernatant of tissues was calculated according to the method reported by Aebi [20].

Ten microliter homogenates were added in 635 µL of phosphate buffer (Na_2_HPO_4_, 0.02 M) and 0.335 µL of H_2_O_2_ (0.03 M). The enzymatic reaction was measured at 240 nm in catalysis mode for 5 min of the reaction (spectrophotometer Genesys 10 S from Thermo Scientific, Waltham, Massachusetts, USA). Catalase activity was calculated as follow:

$${\text{kat}} = {\text{ln }}\left( {{\text{Abs}}{_1}/{\text{Abs}}{_2}} \right)/\Delta {\text{t}}.$$

where, Δt, is the difference between times t_2_ and t_1_. Was reported kat/mg protein.

### Reduced glutathione (GSH)

Twenty microliter homogenates were added in 0.610 mL Na_2_HPO_4_ phosphate buffer (0.3 M, pH 7.2) and DTNB (0.04% in 1% citrate). The solution was incubated for 5 min at room temperature. After centrifuging at 10,000 rpm for 5 min, the supernatant was collected and read at 412 nm. Results were extrapolated in a standard glutathione curve and reported in nmol/mg protein [21].

### Lipoperoxidation

The final products concentration of lipoperoxidation were determined by thiobarbituric acid reactive substances (TBARs), represented as malondialdehyde (MDA). MDA was determined in homogenate tissue with phosphate buffer (pH 7.2) at which the protein concentration was determined. An aliquot of 200 mL was taken which was precipitated with TCA (5% w/v) and was centrifuged at 4000 rpm for 7 min. An aliquot of 100 µL of the supernatant was taken and 0.8% w/v TBA reagent solution (dissolved in 0.026 M Tris-HCl) and 0.6 N of HCl was added (final volume 1200 µL). After 20 min in a boiling bath a color developed. They were read by spectrometry at a wavelength of 535 nm. In addition, a standard curve was prepared with TEP (2.2–22 ng), which is equivalent to 0.72–7.2 ng MDA. The results were extrapolated to the curve and reported in ng/mg of protein previously determined [22].

### Protein quantification

Protein was determined by the Lowry colorimetric method [23]. All the analyses were determined using a Spectrophotometer (JENWAY 6315).

### Statistical analyses

The results from first experiment were analyzed by Student´s T-test and were expressed as mean ± SEM, with a p < 0.05. To compare the results of the second experiment, the Kruskal-Wallis test with multiple comparisons was used. They were expressed as mean ± SEM, with p < 0.05. Non-parametric prove of Correlation Spearman for association of variables (MDA vs. GSH and CAT vs. MDA) was used, with p < 0.05. All proves were evaluated using GraphPad Prism software v. 6.

## Results

### Experiment I. Effect of 14 days of CUMS on brain oxidative balance

Catalase activity, reduced glutathione, and malondialdehyde concentration were used as oxidative balance parameters. There were no significant differences in any of these oxidative balance parameters between CUMS14 and C14 groups analyzed on the cerebellum (Table 2).

**Table 2:** Effect of CUMS at 14 days on biomarkers of oxidative balance in the cerebellum

Cerebellum	C14	CUMS14
CAT (kat/mg protein)	0.16 ± 0.02	0.19 ± 0.02
GSH (nmol/mg protein)	22.76 ± 5.57	26.24 ± 9.54
MDA (ng/mg protein)	87.31 ± 5.14	78.26 ± 10.46

The cerebrum of rats exposed to CUMS for 14 days showed a significant difference, with greater lipoperoxidation than of the control group (Table 3). No change in catalase activity or reduced glutathione levels in both groups was observed (p > 0.05).

**Table 3:** Effect of CUMS at 14 days on biomarkers oxidative balance in the cerebrum

Cerebrum	C14	CUMS14
CAT (kat/mg protein)	0.11 ± 0.01	0.11 ± 0.01
GSH (nmol/mg protein)	24.82 ± 8.26	29.85 ± 10.18
MDA (ng/mg protein)	49.51 ± 4.71	70. 94 ± 9.59*

*p < 0.01 (Student's T-test)

### Experiment II. Stress parameters

**Fig. 1:**
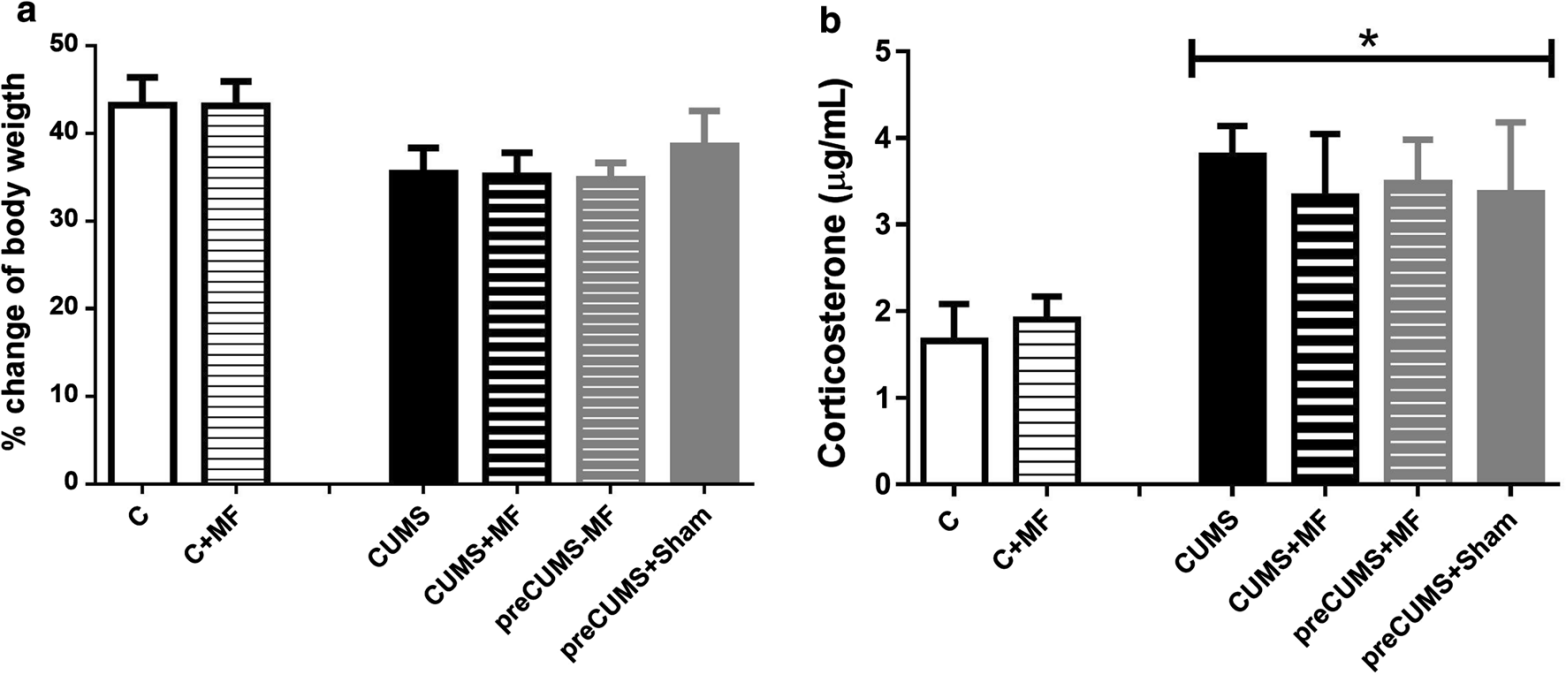
Stress parameters. **a** Percentage of weight gain; **b** Plasma corticosterone concentration. Each column represents mean ± SEM (n = 8). The data were analyzed by the Kruskal-Wallis test with multiple comparisons. *p < 0.05 between all CUMS groups vs. control group

Animals submitted to stress exposition significantly increase the corticosterone concentration compared to control groups with p = 0.0187 (Fig. 1b). No significant differences were observed in the weight gain after the treatments (Fig. 1a).

### Effect of 21 days CUMS and ELF-MF exposure on brain oxidative balance

**Fig. 2:**
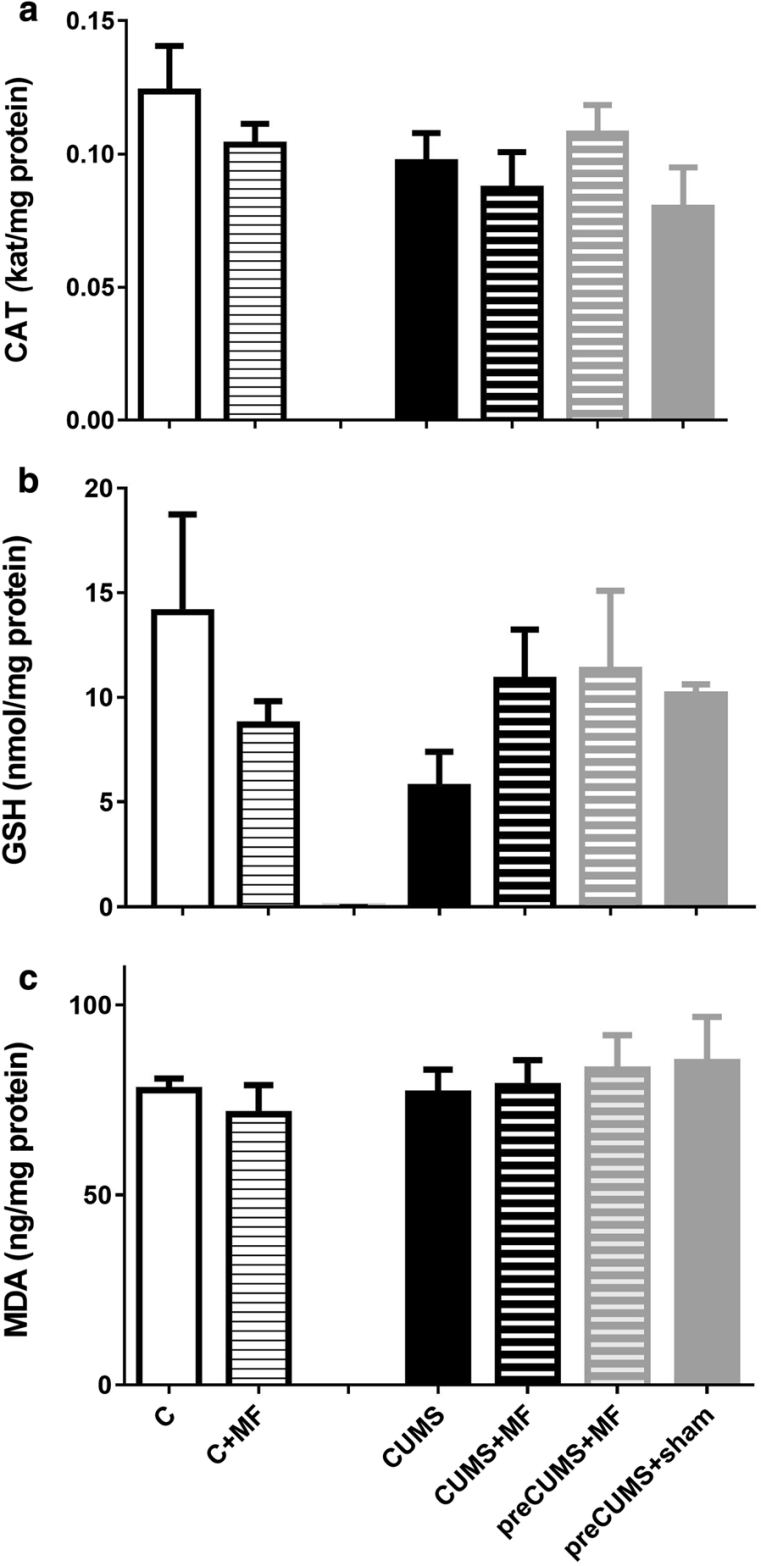
Biomarkers of oxidative balance in the cerebellum. **a** Catalase (CAT kat/µg protein); **b** reduced glutathione (GSH nmol/µg protein); **c** malondialdehyde (MDA ng/mg protein). Data are expressed as mean ± SEM (n = 8)

The biomarkers of oxidative balance measured in the cerebellum are shown in Fig. 2. There was no statistical difference in these parameters in the cerebellum.

**Fig. 3:**
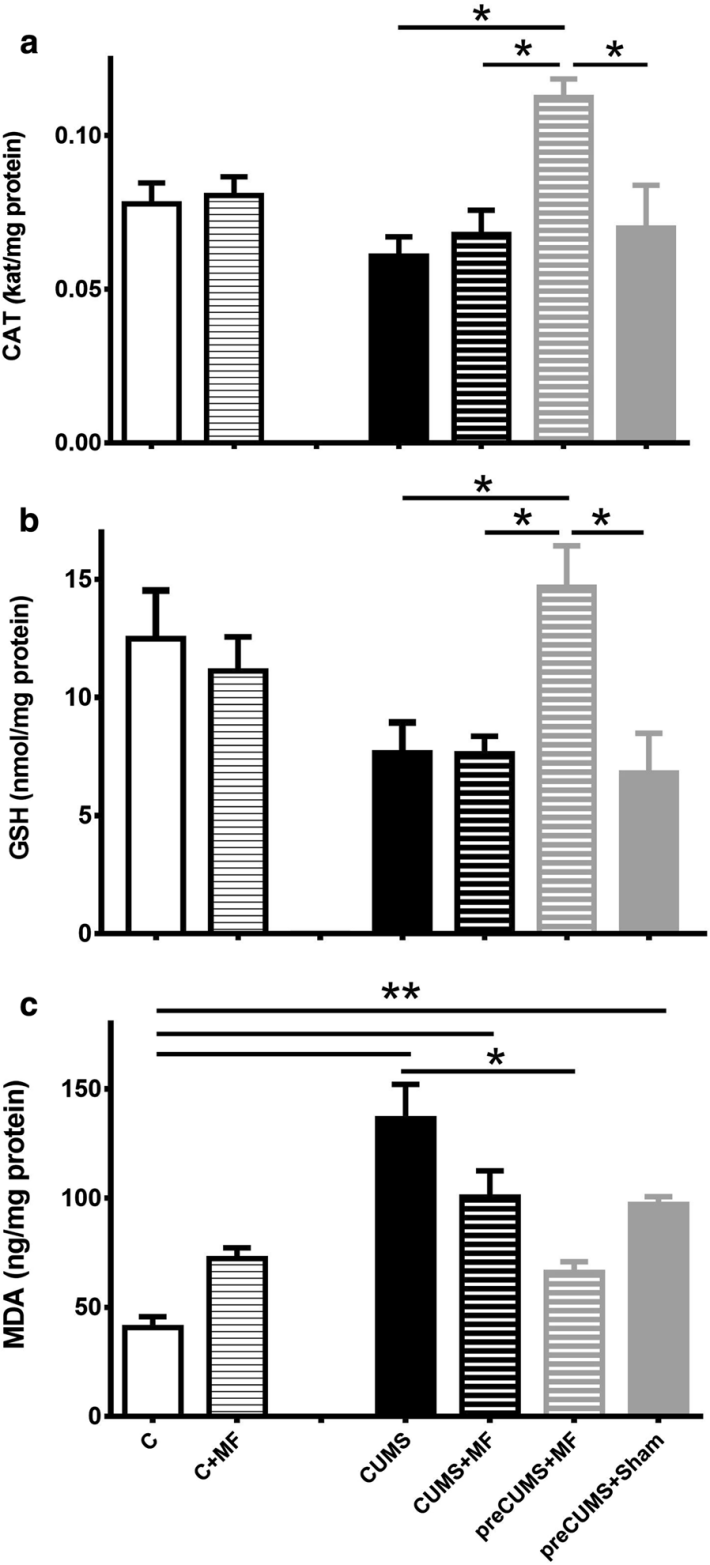
Biomarkers of oxidative balance in the cerebrum. **a** Catalase (CAT kat/mg protein); **b** reduced glutathione (GSH nmol/mg protein); **c** malondialdehyde (MDA ng/mg protein). Data are expressed as mean ± SEM (n = 8). The data were analyzed by the Kruskal-Wallis test with multiple comparisons. *p < 0.05, **p < 0.01

Figure 3 shows three biomarkers of oxidative stress measured in the cerebrum of rats. The catalase activity and GSH concentration significantly increase in the preCUMS + MF group compared to the other stressed groups (p < 0.05, Fig. 3a, b). There was significantly greater oxidation in CUMS, CUMS + MF, and preCUMS + Sham groups compared to the C group, expressed as higher levels of MDA (Fig. 3c). It is important to note that the preCUMS + MF group significantly reduced the MDA concentration compared to CUMS + MF group (Fig. 3c). In this same group -which showed less oxidation- significantly elevated both the catalase activity and the GSH level, compared to CUMS + MF group was observed.

The correlation of Spearman (r) was evaluated for MDA vs. GSH and MDA vs. CAT. A correlation coefficient r = 0.5787 with a p = 0.0038 and r = 0.3767 with p = 0.0152 respectively, were found.

## Discussion

The data of the present study showed that two weeks of CUMS induced oxidative damage in the rat´s cerebrum. Magnetic field exposure was able to reduce the oxidation elicited by the stressful condition when the magnetic treatment was applied after removing the CUMS (preCUMS + MF). The change in oxidative balance was not observed when the rats were at the same time exposed to stress and MF (CUMS + MF).

### Effect of 14 days of CUMS on brain oxidative balance (experiment I)

One of the best biomarkers of oxidative damage is the MDA. It has been considered a measure of lipoperoxidation by many authors [22, 24]. We showed the effect of 14 days CUMS exposure as an increase of MDA in cerebrum but not in the cerebellum. A previous report showed an increase of MDA in the hippocampus and cerebrum when rats were deprived of sleep for 24 h [25]. However, they did not find an increase in other oxidative biomarkers, as in the present study.

### Experiment II. Stress parameters

Some authors considered that ELF-MF exposure acts as a mild stressor in organisms [9, 14]. We found elevated plasma corticosterone concentration in Wistar rats under stress conditions like in other reports [26]. No changes in plasma corticosterone were induced by MF exposure in any condition. Seven days of MF exposure did not induce elevation of corticosterone. The results are similar to previous reports, in which the plasma corticosterone concentration does not vary due to different types of MF exposures [27–30].

Chronic stress exposure induces a decrease in the weight of animals [31, 32]. However, in our study, the 21 days of CUMS exposure did not reduce the percentage of weight gain. Also, the ELF-MF exposure did not affect weight gain in any of the groups, regardless of whether they are non-stressed or stressed animals, similar to previous reports [14]. The MF exposure used in our study does not appear to induce stress in animals.

### Effect of CUMS and ELF-MF exposure on brain oxidative balance

The effect of MF exposure on the formation of reactive oxygen species (ROS) in the brain is variable. Some reported ROS induction [12, 33–36], others found no changes [37, 38], or reduction [39].

Glutathione is an endogenous antioxidant and a cellular defense agent against oxidative damage. Physiological stress is related to a decreased level of GSH in brain tissue [40]. In the present report, the GSH concentration showed a decrease in stressed animals, which confirms that the CUMS model provokes deterioration in this antioxidant system. However, this decrease was only compared to the preCUMS + MS group. Besides, the exposure to ELF-MF does not alter the GSH level on unstressed animals. In the preCUMS + MF group the main effect was an increase in GSH concentration. The reports about the effect of MF on the glutathione system are variable. The long term MF exposure on the rat brains has shown a significant decrease in GSH [33]. In contrast, MF exposure (4 days) induced elevation of the GSH level in the guinea pig brains [41], similar to our result. Coskun, proposes that MF exposures affect various tissues distinctly because they have different tissue antioxidant status and responses.

Some authors propose that the mechanism of action of MF exposure is the formation of free radicals and the impairing of the antioxidant system [8]. This uncontrolled increase in ROS may result in lipid peroxidation [42]. The induction of oxidative damage by MF exposure in unstressed animals has been previously reported [43]. In our study, animals that were not stressed plus MF presented less lipoperoxidation than other groups. The difference between these results may be due to the time of MF since they chronically exposed their animals (24 h/day/7 days).

The CUMS induced an increase in cerebrum lipoperoxidation in all groups, except in the preCUMS + MF group. In this group, a significant reduction in MDA concentration was observed. Very few studies have reported the reduction of oxidative damage induced by MF exposure [44–46]. Recently, Wang and colleagues showed the effect of pulsed EMF treatment in a model of spinal cord injury on Wistar rats. The treatment reduces inflammation, oxidative damage, and promotes functional recovery in the model [46]. Ischemic stroke patients treated with extremely low-frequency MF improved activity and reduced plasma oxidative damage [44]. Seifirad et al., showed that single exposure (60 Hz and 0.5 mT) significantly increased lipid peroxidation on serum. They found an irreversible oxidative imbalance in chronic stimulation [47]. Therefore, these reports and our results allow us to propose that the antioxidant response of tissues to electromagnetic stimulation depends on the exposure parameters and the metabolic state of the organisms.

The CUMS + Sham group allows us to rule out the natural recovery after stopping stress and leaving the animals without any treatment. In the animals of this group, lipoperoxidation did not decrease. Besides, the increase in GSH concentration and catalase activity was not observed in the brain. Then, the increase in the antioxidant system and the lipoperoxidation reduction in the preCUMS + MF group is due to MF exposure.

Spearman correlation showed an inverse relation between MDA measurements and GSH. When there is depletion of GSH in the inside of the cell, there is lipoperoxidation, because there is much less of this antioxidant mechanism, and vice versa. There is also an inverse relation between MDA measurements and CAT activity. These results could be explained by the following proposed mechanism: ROS such as superoxide, hydroxyl ions, and peroxyl radicals cause injury in the brain when they are in excess, and the antioxidant defense is impaired. Continuous stress produces an excess of hydrogen peroxide. Thus, it can follow the route of the Fenton reaction producing an increase of hydroxyls, peroxides and therefore more lipoperoxidation. But in the preCUMS + MF animals, the magnetic exposure induces the increase of catalase activity that could lead to degrading the peroxide of hydrogen as much as possible (“protector shoot”), and therefore it avoids higher concentrations of MDA. The increase of CAT activity could stabilize and improve the reducing system increasing the concentration of glutathione. Another possible mechanism is the hormesis phenomenon, it has been proposed the redox status represents a form of it, characterized by beneficial biological effects at low levels of ROS production and harmful outcomes at high steady-state concentrations [48]. So, the magnetic field exposure applied after CUMS, could act as mild second stress, and allow the brain to lead to an antioxidant response, which could be beneficial. Both hypotheses should be explored in future experiments.

## Conclusions

Seven days of magnetic field exposure reduces the cerebral oxidation elicited by the stressful condition, only if the magnetic treatment is applied after removing the CUMS. The extremely low-frequency electromagnetic field exposure can partially restore the cerebrum antioxidant system of previously stressed animals.

## Limitations

Our study has some limitations to consider in upcoming experiments. Firstly, we used only adult male animals. Female rats were found to be more vulnerable to chronic mild stress [49]. Future experimental studies are needed to elucidate the role of gender on the response of the brain’s oxidative balance to EMF exposure. Secondly, our study did not fully explore the cellular mechanism to explain the protective effects observed. Different modulating mechanisms in addition to the oxidative balance presented in this work may also be involved, like changes in the immune system [50] or protection from apoptosis [51]. Thirdly, the magnetic flux density used in this work was 2.4 mT, like the use of magnetic therapy [3, 52]. Further work that includes other magnetic flux density is recommended.

## Data Availability

The datasets generated and/or analyzed during the current study are not publicly available due to my work privacy institutional commitments but are available from the corresponding author on reasonable request.

## Abbreviations

C: Control
C + MF: Control + magnetic fields
CAT: Catalase
CUMS: Chronic unpredictable mild stress
CUMS + MF: Chronic unpredictable mild stress + magnetic fields
DNA: Deoxyribonucleic acid
ELF-MF: Extremely low-frequency magnetic fields
ELISA: Enzyme-linked immuno sorbent assay
GSH: Reduced glutathione
MDA: Malondialdehyde
mT: Milli Tesla
preCUMS + MF: Chronic unpredictable mild stress + magnetic fields
preCUMS + Sham: preChronic Unpredictable Mild Stress + Sham
ROS: Reactive oxygen species
SEM: Standard error of mean
TBARs: Thiobarbituric acid reactive substances
